# The ubiquitin proteasome system in Huntington's disease and the spinocerebellar ataxias

**DOI:** 10.1186/1471-2091-8-S1-S2

**Published:** 2007-11-22

**Authors:** Janet E Davies, Sovan Sarkar, David C Rubinsztein

**Affiliations:** 1 Department of Medical Genetics, Cambridge Institute for Medical Research, Wellcome/MRC Building, Addenbrooke's Hospital, Hills Road, Cambridge, CB2 2XY, UK

## Abstract

Huntington's disease and several of the spinocerebellar ataxias are caused by the abnormal expansion of a CAG repeat within the coding region of the disease gene. This results in the production of a mutant protein with an abnormally expanded polyglutamine tract. Although these disorders have a clear monogenic cause, each polyglutamine expansion mutation is likely to cause the dysfunction of many pathways and processes within the cell. It has been proposed that the ubiquitin proteasome system is impaired in polyglutamine expansion disorders and that this contributes to pathology. However, this is controversial with some groups demonstrating decreased proteasome activity in polyglutamine expansion disorders, some showing no change in activity and others demonstrating an increase in proteasome activity. It remains unknown whether the ubiquitin proteasome system is a feasible therapeutic target in these disorders. Here we review the conflicting results obtained from different assays performed in a variety of different systems.

**Publication history:** Republished from Current BioData's Targeted Proteins database (TPdb; ).

## Protein pathway in disease

Nine neurodegenerative disorders are caused by the abnormal expansion of polyglutamine-encoding CAG repeat sequences: Huntington's disease (HD), spino-cerebellar ataxia (SCA) types 1, 2, 3, 6, 7 and 17, spinobulbar muscular atrophy and dentatorubral-pallidoluysian atrophy [1]. These disorders are generally inherited in an autosomal dominant manner (except for spinobulbar muscular atrophy, which is X-linked) and the underlying mutations are thought to act predominantly via toxic gain-of-function mechanisms [2]. Mutant proteins with expanded polyglutamine tracts form aggregates in the affected tissues [3-5]. Recently, polyglutamine-containing aggregates were reported in SCA 8 [6]. SCA 8 is caused by the expansion of a CTG repeat in the 3' end of the ataxin 8 gene, which was previously thought to be untranslated. The polyglutamine-containing protein associated with SCA 8 is now thought to be encoded by a previously unidentified antiparallel transcript spanning the repeat in the CAG direction [6].

HD and many of the dominant SCAs are caused by the expansion of a polyglutamine tract within the coding region of the huntingtin and ataxin genes, respectively. Although HD and the SCAs are caused by single mutations (the expansion of a CAG tract), the expression of a mutant protein containing a polyglutamine expansion is thought to have a variety of cellular consequences and elicit many pathogenic mechanisms. For example, in HD, mutant huntingtin has been proposed to cause the dysregulation of transcription [7], either through the interaction of transcription factors (e.g. CBP) with mutant huntingtin or by the polyglutamine expansion disrupting wild-type huntingtin's role in regulating transcription [8,9]. Wild-type huntingtin has been shown to bind repressor element-1 silencing transcription factor/neuron-restrictive silencer factor (REST/NRSF), sequester it in the cytosol and reduce its inhibitory effect on promoters containing neuron-restrictive silencing elements such as the BDNF promoter, thus increasing the production of BDNF. This control is lost in HD; there is a reduced interaction between mutant huntingtin and REST/NRSF, leading to a reduction in the production of BDNF [8,9]. Expression of mutant huntingtin has also been shown to cause excitotoxicity and damage mitochondria, leading to the increased generation of destructive oxygen free radicals and to alterations in energy production [10], disruption of transport along axons [11] and impairment of endocytosis and intracellular vesicle transport [12]. Although controversial, mutant huntingtin has also been proposed to impair the ubiquitin proteasome system (UPS) [13-15]. The polyglutamine expansion mutation is thought to cause disruption of the same systems and elicit similar pathogenic mechanisms in SCA [16].

The UPS is a major protein degradation pathway in cells, typically degrading short-lived and damaged proteins [17,18]. The proteasome also has a role in cell signalling, as it degrades many regulatory proteins, such as p53 and IKKB, and protein subunits. Recently, it was proposed that the proteasome has a role in normal synaptic function and plasticity, and is involved in the NMDA-dependent remodelling of the protein composition of synapses [19]. Therefore, the proteasome not only represents a degradation pathway but is also a major regulator of normal cellular and physiological functioning. Impairment of the UPS will thus have considerable consequences on the cell and indeed the organism.

Two mechanisms have been proposed to account for the UPS impairment observed by some groups in polyglutamine expansion disorders. The first arises from the observation that polyglutamine aggregates in cell models, brains from HD transgenic mouse models and HD post mortem brains become labelled upon immunocytochemistry with antibodies against ubiquitin and proteasome subunits [3,20,21], suggesting that the sequestration and altered subcellular localisation of UPS components could impair its normal function. However, inhibition of the proteasome has been demonstrated in cells co-expressing a GFP–degron construct (see Models, Knockouts and Assays) and pathogenic huntingtin exon 1 constructs in the absence of visible aggregates [14]. In addition, there is some evidence to suggest that some molecules are not sequestered tightly into aggregates but are only loosely associated and can freely diffuse [22]. The second model comes from both *in vitro* and cell model data suggesting that expanded polyglutamine-containing proteins are not easily degraded by the eukaryotic proteasome, which can only accommodate unfolded proteins [23,24]. This model proposes that proteins containing expanded polyglutamine tracts may block the proteasome, thus preventing the entry of other substrates. Although it has been documented that synthetically generated polyglutamine aggregates do not inhibit 26S proteasome function *in vitro*[14], it has recently been shown that fibrillar species purified from HD transgenic mouse and human HD post mortem brains do decrease proteasome activity *in vitro*[25]. In addition, many of the wild-type ataxins have been shown to interact with components of the UPS. Yeast two-hybrid assays have demonstrated an interaction between ataxin-7 and the S4 subunit of the 19S proteasome [26], ataxin-1 and the ubiquitin-like protein A1Up [27], and ataxin-3 and the ubiquitin and proteasome binding factor HHR23B [28]. It has been proposed that these wild-type ataxins have a role in UPS function; thus it is possible that expansion of the polyglutamine repeats in these proteins may disrupt interactions with the UPS and compromise its function.

The impairment of the UPS in polyglutamine expansion disorders is controversial since some groups demonstrate a decrease in proteasome activity [13-15,29,30], some show no change in activity [31,32] and others demonstrate an increase in proteasome activity [33,34]. These conflicting results come from different assays performed in a variety of different systems [35] (which may indeed represent different stages of disease) and are discussed in more detail below.

## Models, knockouts and assays

A variety of approaches have been used to study UPS function in polyglutamine expansion disorders (see Table 1) [36]. Each assay has advantages and disadvantages, and could be monitoring a different part of the UPS pathway. In addition, these assays have been used to assess UPS activity in different models (stable, inducible and transient cell models, transgenic *Drosophila*, transgenic mice and human post mortem samples, see Table 2). These models could represent different stages of the human disease, express the polyglutamine-containing protein at different levels under the control of different promoters, and in the case of Huntington's disease models, express huntingtin transgenes of different sizes (e.g. full length huntingtin or smaller exon 1 fragments).

**Table 1:** Assays used to study UPS function in polyglutamine expansion disorders. See text for further details.

**Method**	**Measures**	**UPS component assayed**	**Advantages**	**Disadvantage**	**Reference**
Fluorogenic substrate peptides	20S proteasome activity	Direct measure of chymotrypsin-like, trypsin-like or peptidyl-glutamyl activity of the 20S proteasome	Quantitative analysis of proteolytic activity in cell and tissue lysates	Does not measure ubiquitylation, substrate interaction, unfolding or effects on other components of the UPS other than proteasome activity	[14, 15, 25, 30, 32, 33]
Degron-tagged fluorescent proteins	Levels of fluorescent reporter protein tagged with a signal targeting it for proteasome degradation	Proteasome activity and targeting to proteasome	Functional analysis of UPS system in vivo	Does not measure all aspects of UPS function	[13, 14, 29, 37, 38]
Levels of endogenous UPS substrates	Degradation of well characterised, endogenous UPS substrates e.g. p53	Ubiquitylation, proteasome activity, chaperones	Functional analysis of entire UPS system. Substrate expressed at endogenous levels	Levels of endogenous substrate may be altered due to effects of the polyglutamine expansion independent of the UPS	[15]
Yeast two-hybrid assay	Interaction of polyglutamine-containing proteins with UPS components	Proteasome subunits and components that interact with polyglutamine proteins	Shows direct interactions	Does not give functional data	[26-28]
In vitro assay of proteasome activity	Effect of synthetic peptides, purifed aggregates and fibrillar species on activity of purified proteasomes	Activity of purified proteasomes	Shows direct effects of poly-glutamine containing proteins on proteasome activity	Does not measure other components of the UPS	[14, 23-25]

**Table 2:** Model systems used to study UPS function in polyglutamine expansion disorders. See text for further details.

**Model**	**Methods used in conjunction**	**Additional notes**	**Reference**
Human post mortem brain	Immunocytochemistry, assay of proteasome activity in lysates using fluorescent substrates		[3, 20, 30]
Human patient skin fibroblasts	Proteasome activity using fluorescent substrates		[30]
R6/2 transgenic mouse model of HD	Proteasome activity, immunocytochemistry,	R6/2 mouse generated by Mangiarini et al. [60]	[21, 34]
R6/1 transgenic mouse model of HD	Proteasome activity	R6/1 mouse generated by Mangiarini et al. [60]	[15]
HD94 conditional mouse model of HD	Chymotrypsin-like, trypsin-like or peptidyl-glutamyl activity in lysates	HD94 conditional mouse generated by Yamamoto et al. [61]	[33]
SCA 7 knock-in mouse model	Crossed to a transgenic mouse expressing an EGFP–degron reporter	SCA 7 transgenic mouse generated by Yoo et al. [62]	[31]
Transgenic Drosophila models of polyglutamine expansion disorders	Genetic screens		[56]
Cell models of HD and SCA, stable cell lines and transient expression of mutant constructs. Inducible or constitutive transgene expression	Immunocytochemistry, assay of proteasome activity in lysates using fluorescent substrates, co-expression of construct with fluorescent–degron reporter.		[13-15, 20, 29, 32]
C. elegans models	Co-expression of mutant ataxin-3 and a ubiquitin-conjugated dsRed reporter		[37]

Proteasome activity can be monitored by the proteolysis of small fluorogenic substrates specific for the chymotrypsin-like, trypsin-like or peptidyl-glutamyl activity of the 20S proteasome. These have been used to measure activity in lysates from neuro2A cells stably expressing N-terminal huntingtin with an expanded polyglutamine tract [15], SH-SY5Y stably expressing polyglutamine–green fluorescent protein constructs [32], transgenic mice [33], human HD post-mortem brains and patient skin fibroblasts [30]. These peptides have also been used to measure the activity of purified 26S proteasomes incubated with either synthetic polyglutamine-containing proteins [14] or aggregates and filaments purified from transgenic mice and human brains [25]. One issue with assays of isolated proteasome activity is that modest changes in proteasome number/activity may not be rate-limiting for substrate clearance. It is likely that ubiquitin conjugation and, in some situations, transport of ubiquitylinated proteins to the proteasome could be more important physiological regulators.

This problem has been partially addressed by measuring proteasome activity using reporter molecules comprising a fluorescent protein (e.g. enhanced green fluorescence protein (EGFP)) fused to a short sequence that targets the protein for proteasome degradation (termed a degron), [13,14,29,37,38]). This approach was initially used to generate a HeLa cell line stably expressing a reporter consisting of a short degron, CL1, fused to the carboxy-terminus of EGFP [13]. More recently, transgenic mice expressing EGFP tagged to a degron [38] and *Caenorhabditis elegans* expressing a ubiquitin-conjugated dsRed reporter [37] have been generated. It is also possible to monitor activity of the UPS by quantifying the levels and the degradation of well known proteasome substrates such as p53 [15].

## Disease targets and ligands

The idea that the UPS may be impaired in polyglutamine expansion disorders initially came from studies showing the labelling of polyglutamine aggregates with antibodies raised against ubiquitin and proteasome subunits in cell models [20,39], transgenic mice [21] and human post mortem samples [3]. It was suggested that the sequestration of UPS components in aggregates and the altered subcellular localisation of proteasomes could affect UPS activity. The first study to assess proteasome activity used fluorogenic substrates [15]. A shift in chymotrypsin-like activity was demonstrated from cytosolic fractions to aggregate-containing, precipitated fractions derived from lysates from both a stable HD cell model (expressing huntingtin exon 1 with a 150 polyglutamine repeat) and brain lysates derived from R6/1 mice (expressing exon 1 of the huntingtin gene with a (CAG)_116_ repeat expansion under control of the huntingtin promoter) [15]. Chymotrypsin-like activity was reduced in the cytosolic fraction and increased in precipitated fractions derived from these lysates when compared with control cells. This suggested the altered localisation of proteasomes to aggregates. The authors also demonstrated reduced degradation of p53. This study suggested an impairment of the UPS in HD. Soon after, this data was further supported by a study using an EGFP–degron reporter [13]. When this reporter was co-expressed with mutant huntingtin in cells, GFP fluorescence was increased more than two-fold when compared with cells expressing wild-type huntingtin. This observation implies a major impairment of proteasome function since a greater than 50% decrease in chymotrypsin-like activity is required to obtain a 50% increase in GFP fluorescence [13]. Similar results were found with the Δ508 mutant cystic fibrosis membrane conductance regulator (a protein that is unrelated to mutant huntingtin and the ataxins, sharing only the propensity to aggregate), suggesting that proteasome impairment is caused by aggregate formation [13]. Similarly, an EGFP reporter containing PEST sequences that target cytosolic proteins for proteasome degradation has been used to monitor proteasome activity in cells expressing ataxin-1 constructs [29]; the polyglutamine expansion mutation in ataxin 1 reduced activity of this assay. Consistent with these data, a reduction of chymotrypsin-like and peptidyl-glutamyl activity has been demonstrated in lysates from human HD post mortem brains and HD patient skin fibroblasts [30]. Similarly, inhibition of proteasome activity by ataxin-3 with an expanded CAG tract has been demonstrated in *C. elegans* using an EGFP–degron reporter [37].

Using the co-expression of NLS- or NES-tagged EGFP–degrons and NES or NLS mutant polyglutamine constructs, a global impairment of the UPS was demonstrated, regardless of the intracellular locations of the proteins containing the expanded polyglutamine tracts or the degron reporters [14]. Bennett *et al.* also disproved two hypotheses proposing mechanisms for the inhibition of proteasome activity. Contrary to the sequestration hypothesis, they demonstrated proteasome inhibition in the absence of visible aggregates. They also showed that synthetic protein aggregates do not inhibit activity of the 26S proteasome function *in vitro* suggesting that UPS impairment is unlikely to be caused by blocking of the proteasome. Indeed, the decreases in nuclear proteasome function by extra-nuclear mutant polyglutamine and *vice versa* argued that the observed effects were independent of interactions between mutant protein and the proteasome. However, it has recently been demonstrated that, whilst aggregates do not inhibit the proteasome, fibrillar forms of huntingtin purified from transgenic mouse and human post mortem brains do inhibit the 26S proteasome *in vitro *[25].

Data contrary to the above, suggesting that the proteasome is not impaired in polyglutamine expansion disorders, comes from a variety of sources. SH-SY5Y cells stably expressing mutant huntingtin do not show a difference in the degradation of fluorogenic peptides, compared with cells expressing wild-type huntingtin [32].

More recently, an increase in the chymotrypsin-like and trypsin-like activities of the proteasome was observed in lysates derived from the cortex and striatum of the HD94 conditional mouse model of HD [33]. This was attributed to an increase in the levels of the proteasome subunits LMP2 and LMP7, and to the induction of the immunoproteasome. Immunoproteasomes are generally induced as part of the immune response (i.e. by interferon-gamma) and possess subunit composition and cleavage specificity favouring the production of peptides suitable for antigen presentation. Increased proteasomal chymotrypsin-like activity has also been observed in brain lysates from the R6/2 model of HD in comparison to non-transgenic littermates [34]. However, this study also demonstrates no change in overall 26S proteasome activity and shows that the nuclear proteasome activator REGγ is not involved in polyglutamine pathology, thus discounting it as a therapeutic target [34].

One of the caveats of studies in cell culture is that they express artificially high levels of mutant proteins. The role of the proteasome *in vivo* has recently been tested using the knock-in mouse model of SCA 7 crossed to a transgenic mouse expressing an EGFP–degron reporter [31]. Bowman *et al.* observed an increase in levels of the reporter in neurons at late stages of the disease. However, this was not due to inhibition of proteasome activity but correlated with an increase in mRNA encoding the EGFP–degron reporter. This study raises the important point that one should check that transcription of this *in vivo* reporter is not perturbed when its steady state level changes, otherwise one may in certain instances incorrectly ascribe accumulations to proteasome dysfunction.

It must be ascertained whether UPS activity is impaired in polyglutamine expansion disorders before the UPS is proposed as a therapeutic target. However, two groups have filed patents on the use of UPS modulators to treat neurodegenerative disorders (see additional file 1 for current patents relating to the UPS in Huntington's disease and the spinocerebellar ataxias). Based on the evidence that the proteasome is compromised in HD, Thompson, Marsh and Steffan have patented methods and reagents for reducing polyglutamine toxicity (no applicant listed), and Ramesh and Sean (ALS Therapy Development Foundation) have patented the use of proteasome modulators to treat neurodegenerative disorders. Lindquist and Duennwald (Whitehead Institute for Biomedical Research) have patented a method of screening for inhibitors of huntingtin-induced UPS impairment.

Mutations in parkin, a ubiquitin E3 ligase, lead to autosomal recessive Parkinson's disease. Parkin deficiency makes post-mitotic neurons more susceptible to excitotoxicty, whilst parkin over-expression protects neurons from kainate excitotoxicity and cell death [40]. Parkin also co-localises with mutant huntingtin aggregates in HD mice and human brains, and overexpression of parkin enhances the clearance of the mutant proteins [41]. Therefore, parkin is a feasible target for both the protection of post-mitotic neurons from excitotoxicity and the treatment of polyglutamine disorders by enhancing the clearance of toxic polyglutamine-containing proteins. Abeliovich and Staropoli (Columbia University) have patented parkin for this use.

There is evidence to suggest that polyglutamine-containing proteins are not efficiently degraded by the proteasome and even directly inhibit activity of the UPS [23-25]. Therefore, glutamine dipeptides, tripeptides or polypeptides have been patented by Goldberg (Harvard University) as bacterial proteasome inhibitors for the treatment of diseases such as those caused by *Mycobacterium tuberculosis. Mycobacterium tuberculosis* is highly resistant to killing by human macrophages, a property thought to be conferred by its proteasome [42]. Thus, inhibiting proteasome activity may attenuate this resistance.

## New frontiers in drug discovery

One strategy for the treatment of polyglutamine expansion disorders is to decrease levels of the toxic mutant protein. This could be achieved by increasing the clearance of the mutant protein. Indeed, induction of autophagy by treatment with the mTOR inhibitor rapamycin has been demonstrated to reduce aggregation and increase survival in HD cell and mouse models [43]. Autophagy is also beneficial in SCA 3 and is generally neuroprotective [44,45].

It is unclear whether proteins with an expanded polyglutamine tract are good proteasome substrates. Huntingtin interacts with the human ubiquitin-conjugating enzyme E2–25K, which requires the polyglutamine domain [46]. As previously described, parkin, an E3 ubiquitin ligase, also co-localises with mutant huntingtin aggregates in HD mice and human brains, and over-expression of parkin enhances the clearance of the mutant protein [41]. These data suggest that huntingtin may be a proteasome substrate. Consistent with this, proteasome inhibitors such as lactacystin and epoxomycin prevent mutant huntingtin clearance in a conditional HD mouse model or cell models after its expression is stopped [47].

Lactacystin, a microbial metabolite, was initially discovered and isolated from actinomycetes on the basis of its ability to induce neurite outgrowth in a murine neuroblastoma cell line [48]. Subsequent work demonstrated that the biological effects of lactacystin resulted from its ability to inhibit the proteasome [49]. Similarly, epoxomycin was isolated from a strain of actinomycetes and shown to exhibit potent anti-tumour and anti-inflammatory effects [50]. The target of epoxomycin was later shown to be the proteasome [51]. An increase in mutant huntingtin aggregation and toxicity in HD cell models after proteasome inhibition has also been demonstrated [15,39,47,52,53]. Likewise, aggregation of polyglutamine-expanded ataxin-1 and ataxin-3 is also increased after proteasome inhibition [54,55]. Furthermore, mutations in the genes encoding ubiquitin, ubiquitin C-terminal hydrolase, ubiquitin conjugase homologous to human UbcE2D2 and ubiquitin conjugase homologous to human Ubc2EH enhance polyglutamine toxicity in *Drosophila* over-expressing mutant ataxin-1 [56]. However, the proteasome is only able to accommodate unfolded proteins and it has been demonstrated that soluble mutant polyglutamine proteins are degraded by the proteasome whereas the aggregated form is resistant to degradation [57]. Likewise, the mutant form of ataxin-1 is more resistant to degradation *in vitro* than the wild-type form, although both the proteins are equally ubiquitylated [55].

Recently, it has been suggested that the proteasome is unable to cleave between glutamine residues within polyglutamine tracts [23,24]. Thus, if one could upregulate proteasome activity, one would possibly reduce the levels of proteins with polyglutamine expansions and associated flanking sequences. However, this would produce increased levels of long isolated polyglutamine tracts and such products are predicted to be more toxic than the inputs that have flanking sequences. Nevertheless, these long polyglutamine tracts are almost certainly degraded by cytosolic and nuclear peptidases, since moderate polyglutamine stretches are not uncommon in mammalian proteomes. It is unclear if the substrate capacity of such peptidases could be overwhelmed if proteasome activity were increased. In addition, modulation of the proteasome may not be a good therapeutic strategy. The proteasome has a key regulatory role and altering its rate of degradation could have many side effects. One may be able to use chemical chaperones such as trehalose or Congo red to increase the degradation of polyglutamine-containing proteins, as these agents shift the equilibrium towards increasing the levels of soluble, monomeric proteasome-accessible species and away from aggregates [58,59].

Further studies are required to resolve the conflicting data on UPS function in polyglutamine expansion disorders. It is possible that more attention should be paid to measuring levels of known endogenous proteasome substrates *in vivo* to test this important hypothesis. The differences in UPS activity seen in different models using a variety of assays must be explained before the UPS is proposed as a therapeutic target in HD and SCA.

## Notes added in proof

Goldberg and colleagues have identified the peptidase downstream of the proteasome that degrades polyglutamine tracts as putomycin sensitive aminopeptidase [63].

In order to try to address if the proteasome is affected by mutant huntingtin *in vivo*, Kopito and colleagues measured levels of polyubiquitin chains as an endogenous biomarker  [64]. The amount of polyubiquitin chains within a cell was shown be a faithful indicator of ubiquitin proteasome system function and elevated levels of polyubiquitin chains were demonstrated in brain lysates from R6/2 HD transgenic mice, the HdhQ150/Q150 knockin model of HD, and human HD post-mortem brains. One question raised by this study is whether the elevated levels of ubiquitin chains are necessarily due to proteasome dysfunction, as opposed to an increase in the ubiquitylation rate of substrates.

## Abbreviations

UPS, ubiquitin proteasome system; HD, Huntington's disease; SCA, spinocerebellar ataxia; EGFP, enhanced green fluorescent protein.

## Competing interests

David Rubinsztein and Sovan Sakar are inventors on patents relating to the use of autophagy upregulation for the treatment of neurodegenerative diseases. The Rubinsztein lab receives some research grant funding from Wyeth who make rapamycins.

## Publication history

Republished from Current BioData's Targeted Proteins database ().

## Supplementary Material

Additional file 1 Current patents relating to the ubiquitin proteasome system in Huntington's disease and the spinocerebellar ataxias. Despite controversy over the role of the UPS in the pathology of HD and the SCAs, several groups have filed patents on the use and screening of UPS modulators to treat neurodegenerative disorders. Whilst it remains to be seen whether UPS activity is impaired in polyglutamine disorders, these patents represent exciting potential therapeutic strategies. Click here for file
