# Supplementary material for: The ubiquitin proteasome system in Huntington's disease and the spinocerebellar ataxias
Source: BMC Biochem. 2007 Nov 22;8(Suppl 1):S2. doi: 10.1186/1471-2091-8-S1-S2 (PMC2106366; doi:10.1186/1471-2091-8-S1-S2)
Supplement: Additional file 1 — Current patents relating to the ubiquitin proteasome system in Huntington's disease and the spinocerebellar ataxias. Despite controversy over the role of the UPS in the pathology of HD and the SCAs, several groups have filed patents on the use and screening of UPS modulators to treat neurodegenerative disorders. Whilst it remains to be seen whether UPS activity is impaired in polyglutamine disorders, these patents represent exciting potential therapeutic strategies. [file 1471-2091-8-S1-S2-S1.doc]

| **Title** | **Patent no.** | **Company** | **Inventors** | Therapy areas | **Actions** | **Technologies** |
| --- | --- | --- | --- | --- | --- | --- |
| Method of screening for inhibitors of Htt-induced impairment of protein degradation. Comprises providing a cell expressing a misfolded Htt protein and a UFD substrate, contacting the cell with a test agent and measuring UFD substrate degradation. | WO-2005108599 | Whitehead Institute for Biomedical Research | Lindquist Susan L.; Duennwald Martin | Huntington’s chorea; spinal muscular atrophy | Neuroprotectant; ubiquitin ligase inhibitor; unspecified regulatory protein modulator | Drug screening; fluorescence |
| Use of glutamine–glutamine dipeptides, glutamine–glutamine–glutamine tripeptides or polypeptides comprising a polyglutamine domain as bacterial proteasome inhibitors - for the treatment of *Mycobacterium tuberculosis* infection. | WO-2005094423 | Harvard University | Goldberg Alfred L. | Bacterial infection; spinocerebellar ataxia; Huntington’s chorea; *Mycobacterium tuberculosis* infection; neurodegenerative disease | Proteasome inhibitor | Peptide |
| Parkin-associated complex for protecting post-mitotic neurons from excitotoxicity and uses thereof. | WO-2005081858 | Columbia University | Abeliovich Asa; Staropoli John Francois | Alzheimer’s disease; Huntington’s chorea; Mmotor neurone disease; Mmultiple sclerosis; myasthenia gravis; neurodegenerative disease; Parkinson’s disease | CNS diagnostic agent; ubiquitin ligase stimulator | Diagnostic method; Ddrug screening; labeling system |
| Methods and reagents for reducing polyglutamine toxicity | US-20040235733 | Individual | Steffan Joan S.; Thompson Leslie M.; Marsh James Lawrence | Alzheimer’s disease; atrophy; spinocerebellar ataxia; diabetes mellitus; epilepsy; Huntington’s chorea; motor neurone disease; neurodegenerative disease; Parkinson’s disease; prion infection; schizophrenia; transmissible spongiform encephalopathy | Ubiquitin ligase modulator |  |
| Use of proteasome modulators for treating neurodegenerative disease | WO-03101481 | ALS Therapy Development Foundation | Ramesh Tennore M.; Scott Sean | Alzheimer’s disease; ataxia; Huntington’s chorea; infection; motor neurone disease; multiple sclerosis; neurodegenerative disease; Parkinson’s disease; prion infection; spinal cord injury; spinal muscular atrophy | Neuroprotectant; protease inhibitor; proteasome modulator |  |
